# Study on the Process Parameters and Corrosion Resistance of FeCoNiCrAl High Entropy Alloy Coating Prepared by Atmospheric Plasma Spraying

**DOI:** 10.3390/ma18071396

**Published:** 2025-03-21

**Authors:** Miao Zhang, Yu Zhang, Pengyu Dai, Lin Zhao, Liping Wu, Shendian Li

**Affiliations:** 1 School of Mechanical Engineering, Shenyang University, Shenyang 110044, China; mzhang@163.com (M.Z.); nihao9985@163.com (Y.Z.); sdli@163.com (S.L.); 2 Institute of Metal Research, Chinese Academy of Sciences, Shenyang 110016, China; lpwu@163.com

**Keywords:** FeCoNiCrAl coating, atmospheric plasma spraying, process parameters, corrosion resistance

## Abstract

FeCoNiCrAl high-entropy alloy (HEA) coating was prepared by air plasma spraying, and the coating’s morphology and properties under different power parameters were analyzed. The results show that the spraying power significantly affects the morphology of the coating during plasma spraying. The molten droplets formed during the preparation process of HEA coatings tend to combine with oxygen, with aluminum bonding particularly strongly with oxygen, resulting in the presence of aluminum oxide within the coating, while other elements exhibit weaker bonding with oxygen. The optimal spraying power is 12 kW, and coatings prepared at this optimal power exhibit advantages such as low porosity, uniform element distribution, and excellent corrosion resistance. The aluminum in the HEA coating forms a relatively stable compound with oxygen, creating a Cr-depleted and Al-enriched region. This region is less prone to passivation during corrosion and more susceptible to reacting with corrosive media, leading to localized corrosion of the coating.

## 1. Introduction

High entropy alloys (HEA) are a special type of alloy composed of five or more elements, with proportions between 5% and 35%, which form a single phase instead of previously expected complex intermetallic compounds seen in conventional alloys, due to high configurational entropy of those alloys. Different types of high-entropy alloys with the special structure have excellent properties in various fields such as corrosion resistance [1,2,3], wear resistance [4,5,6], and magnetic properties [7,8,9], and they have great prospects for development in various industrial production, including energy applications.

There are many methods for preparing high entropy alloys, such as laser cladding [10], sputtering [11], vacuum arc [12] techniques, and thermal spray techniques [13,14]. Among those, the thermal spray technique is one effective method [15,16], which is widely adopted. The process of thermal spraying involves the application of a coating by propelling molten powder onto a surface at velocities achieved through the use of an intense heat source, making it an ideal technique for coating fabrication. Compared to alternative methods, atmospheric plasma spray (APS) has advantages over other spraying methods [17,18], because it is less restricted in material selection, and has higher spray rate and deposition efficiency. During plasma spraying, the plasma beam melts spray powder with minimum preheating and cooling, and spraying conditions can be easily controlled for various applications [19,20]. Therefore, many materials, including metals, metal oxides, and non-metallic materials can be coated using plasma spraying. However, HEA powders are melted by a plasma beam during the spraying process and are susceptible to oxidization within their exposure for a short time. These molten particles impact the substrate and quench, which results in numerous defects such as plats, inter-splat cracks, and porosity in the coatings [21]. Alterations in spraying parameters cause an increased amount of oxide spatter, dendritic morphology, and other defects in the coating, all of which influence the widespread application of plasma high-entropy alloy coatings. Cheng [22] investigated spray parameter variations on the properties of APS-sprayed AlCrFeCoNi HEA coatings. They reported that a fraction of the BCC phase from the powder subsequently transformed to the FCC phase in the coating microstructure after APS spraying. Mu [23] found that the deposition of AlCrFeCoNi coatings resulted in the formation of BCC and oxide phases in the coating structure by APS and rummaged the nanoscaled multiple oxide phases using the XPS method. The melting–solidification process of the sprayed powder during spraying changes the coating structure and properties. Therefore, the influence of spraying parameters, particularly spraying power, on the performance of high-entropy alloy coatings demands further research.

There are various components in high entropy alloys. Among those, FeCoNiCrAl has been widely researched due to its unique competencies in terms of microstructure and mechanical properties [24,25,26] and shows the well corrosion resistance and passivation performance comparable with 316L stainless steel. FeCoNiCrAl high entropy alloys or coating can be used in industrial parts against chemical and mechanical attacks. In FeCoNiCrAl components, Cr/Ni and Co are the base alloying forming a single-phase BCC or chemically, ordered BCC (B2) structure in the equimolar FeCoNiCrAl composition; Al can considerably influence the phase formation of the FeCoNiCrAl alloys [23].

HEA is fabricated using different additions (such as vanadium [27]/titanium [28,29]) or different manufacturing technologies (such as vacuum induction melting/laser cladding [10]), the specimens usually show different properties from thermally sprayed coatings due to the difference in structure and microstructure. Similarly, the coating prepared by the plasma spraying process will produce different performance coatings due to different process parameters, such as the spraying power. The introduction of oxygen elements and oxides during the spraying process, as well as inevitable coating defects, have an impact on the corrosion resistance and other properties of the coating, and exhibit characteristics different from other preparation methods. From the perspective of industrial applications, the influence of preparation methods and process parameters on coating performance constrains the prospects of large-scale application of coatings.

We prepared FeCoNiCrAl high-entropy alloy (HEA) coating by air plasma spraying (APS), and the coating’s morphology and properties under different power parameters were analyzed. The purpose of this study is to find out the influence of the performance and structure of the coating under different spraying powers on the corrosion resistance and passivation performance of the coating and to analyze the role of each element in the corrosion and passivation process.

In this study, atmospheric plasma spray (APS), which involves generating a high-temperature plasma by passing a gas through a high-voltage arc, operates at power levels of 9–18 kW. FeCoNiCrAl alloy coating was prepared by APS, and the effect of spraying power on the structure and properties of the coating was studied.

## 2. Materials and Methods

### 2.1. Sample Preparation

**Figure 1:**
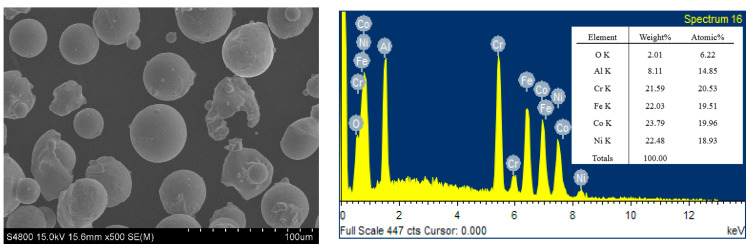
SEM image and elemental mappings of the powder.

The FeCoNiCrAl alloy powder was obtained from Beijing ZhongKe Nuo Company (Beijing, China). The powder has a purity of 99.9% with the molar ratio of various elements in 1:1:1:1:1. The distribution range of powder size is from 15 to 50 microns. The SEM image and elemental mappings of the powder are shown in Figure 1.

The powder should be dried at 60 °C for 24 h before spraying in order to increase its fluidity. The substrate is Q235 steel, with the chemical composition of Q235B (mass fraction, %): C 0.20, S 0.036, P 0.017, Mn 0.58, Si 0.21, Cu 0.02, Fe residual. The Q235B substrate is precision-cut into dimensions of 20 mm by 20 mm by 3 mm by using wire-cutting techniques. The sample was grit-blasted with 80# white corundum sand under pressure of 0.6~0.8 Mpa to achieve a specific level of cleanliness and roughness before spraying, followed by placement in a drying oven for drying and preheating at 60 °C.

### 2.2. Spraying Experiment

The HEA coating was fabricated by using the Guangzhou Sanxin SX-80 plasma spraying system (Guangzhou, China). The main gas was Argon, and the second was Hydrogen, which prevented the oxidation reaction of the powder in the high-temperature melting state during the spraying process. The process parameters of spraying are shown in Table 1 below. Except for spraying power, other spraying parameters were consistent in the study of the effect of spraying power on the coating.

### 2.3. Coating Characterization and Electrochemical Experiments

A type II S-4800 scanning electron microscope (SEM) (Hitachi, Tokyo, Japan) was utilized to examine both the surface and cross-sectional morphology of the coating. Elemental mapping of the coating was conducted with an energy-dispersive X-ray spectrometer (EDS), which is integrated with the SEM system. The crystal structure of the coating was investigated using a Rigaku-D/max 2000 X-ray diffractometer (XRD) (Tokyo, Japan), operating at a current setting of 250 mA with a scanning speed of 2θ per minute.

With the Princeton 2273 electrochemical workstation, the electrochemical experiments of the coating were carried out, including Mott–Schottky measurements. The working electrode(WE) was HEA coating; the counter electrode (CE) was 20 mm × 20 mm × 0.1 mm platinum electrode; the reference electrode (RE) consisted of a salt bridge and calomel electrode filled with saturated KCl solution(SCE), and the solution in the electrolytic cell was 5 wt% NaCl. The potential sweep for the Mott–Schottky analysis spanned from −1.0 V to 1.0 V, performed at a scanning velocity of 10 mV per second and an oscillation frequency of 1 kHz, using a reference electrode for comparative measurements. The estimate of element dissolution in 5 wt% NaCl solution was obtained by using inductively coupled plasma optical emission spectroscopy.

## 3. Results and Discussion

### 3.1. SEM Results of the Coatings

**Figure 2:**
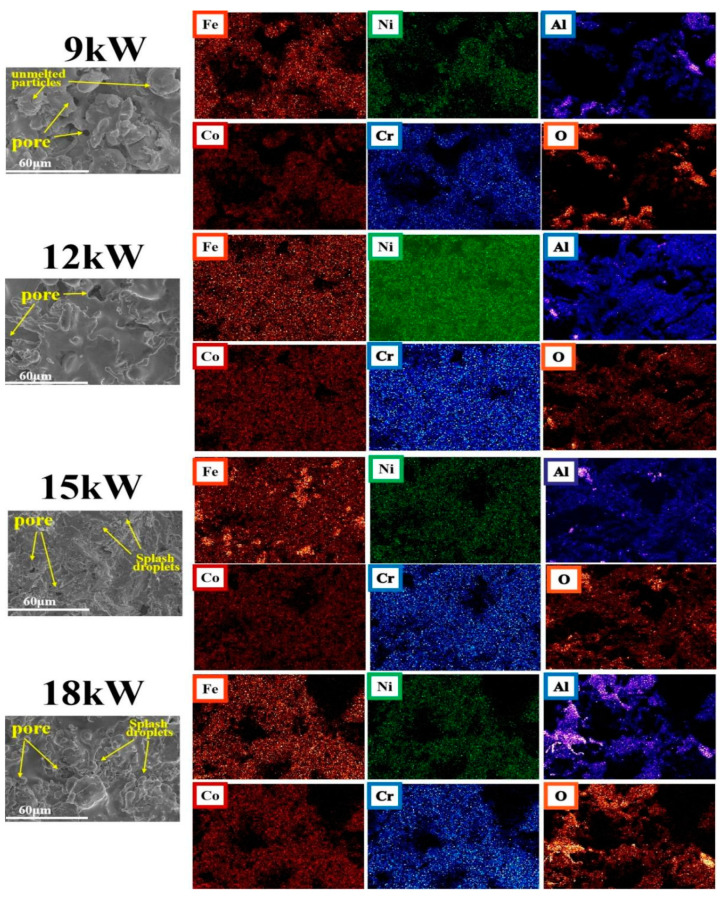
SEM results of FeCoNiCrAl coating surface with different spraying powers and elements distribution mappings on the coating surface.

Figure 2 shows SEM images of FeCoNiCrAl coating surfaces with different spraying powers, as well as element distribution mappings of the coating surface. There are some pores and strip-shaped defects on the surface of coatings with different powers. This is because, during the plasma spraying process, high-temperature molten powder droplets are sprayed onto the substrate surface at lower temperatures along with the main airflow. During the cooling and film formation process, the rapid shrinkage and expansion of the film layer will form defects such as pores and cracks [30]. The element distribution mappings of the coating surface show that the coatings prepared with 9 kW and 18 kW had relatively poor uniformity in element distribution, and local agglomeration of aluminum and oxygen was more obvious.

**Figure 3:**
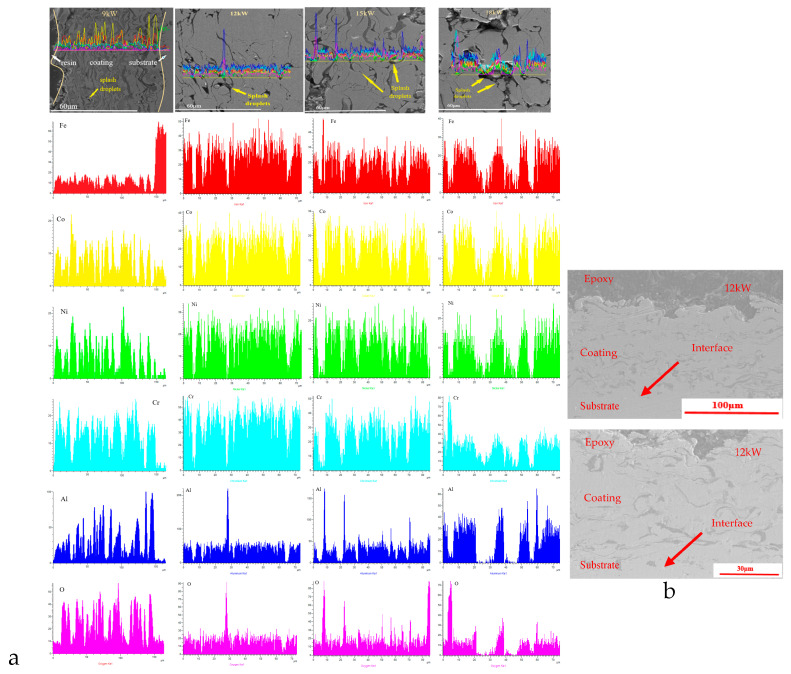
Cross-section SEM results of FeCoNiCrAl coating with different spraying powers on the coating cross-section sides ((**a**) elements distribution mappings; (**b**) cross-section images with 12 kW).

Based on the cross-sectional scanning electron microscopy and line scanning results of the coating shown in Figure 3a, it can be observed that there are significant differences in the surface defects of the coating at different spraying powers. There are a large number of unmelted particles on the surface of the coating with a spraying power of 9 kW, which is because the power of spraying is too low, and some powders have not reached the molten state. During the deposition process, the deformation of molten droplets hitting the substrate is insufficient, and there is a significant amount of powder bouncing loss, resulting in low deposition efficiency and decreased coating quality. With the increase in spraying power, coatings with 12 kW, 15 kW, and 18 kW did not exhibit this phenomenon.

Figure 3b shows cross-section SEM images of FeCoNiCrAl coating with 12 kW, and 15 kW spraying power. It can be found that the coating is well bonded with the substrate without obvious delamination. At the interface between the coating and substrate, there is slight mutual penetration, mainly because both of them have Iron elements, and there will be slight element penetration in the spray process, which makes the well-protective effect of the coating on the substrate.

When the spraying power was increased to 15 kW, some splashing droplets appeared on the surface of the coating due to the high energy of the molten powder droplets hitting the substrate [31], and the number of splashing droplets also increased with the increase in power.

The coating prepared at 18 kW has the highest spraying power, the highest temperature of the molten droplets, the highest activity, and the longest time required for solidification from liquid to solid after reaching the substrate. During the film formation and cooling process, the more active aluminum in the coating oxidizes with oxygen in the external environment, resulting in a higher oxygen content in the mapping of the coating. The cross-sectional morphology shows that the dark aluminum element stripes are more prominent. If the spraying power is too high, the plasma jet temperature will be too high, and the powder particles will be severely overheated or excessively melted, which is not conducive to the coating stacking and bonding with the substrate, forming more pores and oxide bands in the coating, reducing the quality and performance of the coating.

The results show that only the coatings with spray power of 12 kW and 15 kW exhibited a relatively compact layered structure, which resulted in better coating quality compared to coatings prepared with other powers. The distribution map of elements on the coating surface also indicates that the coating surface with a spraying power of 12 kW has the most balanced distribution of all elements.

**Figure 4:**
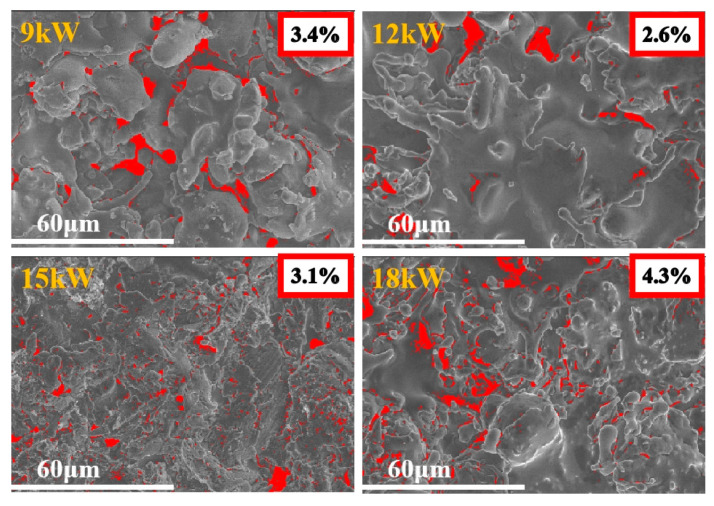
Porosity results of FeCoNiCrAl HEAs coatings with different spraying powers after Image-J processing.

Figure 4 shows the comparison of surface porosity of coatings prepared under different power. Coatings prepared under higher power have higher porosity, and the optimal spraying power is 12 kW.

The element distribution mappings of the coating reveal an uneven distribution of aluminum across all samples, characterized by pronounced clustering. The alignment of oxygen and aluminum distribution patterns suggests that the affinity of oxygen for other elements is comparatively low during the spraying process, with a predominant interaction occurring with aluminum, and existing in the form of aluminum oxide. The Energy Dispersive Spectrometry (EDS) results show that the oxygen concentration in the local area of the dark stripes in the coating can reach 40%, and the aluminum concentration is about 20%, which is similar to the previous findings [29].

Among the coatings prepared at different powers, the coating prepared at 12 kW has a lower relative oxygen concentration distribution and better uniformity of element distribution.

### 3.2. XRD Results

**Figure 5:**
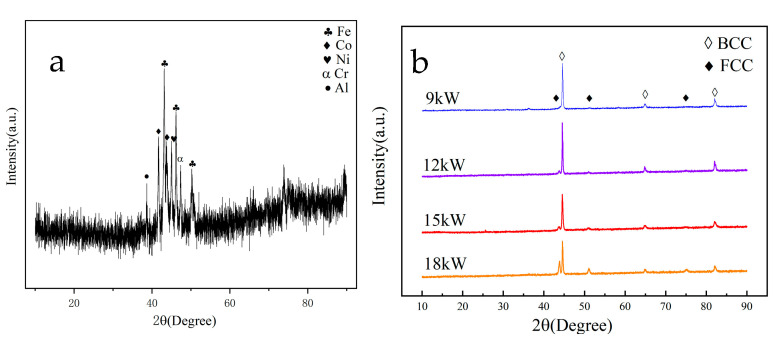
XRD patterns of FeCoNiCrAl coatings with different spraying powers ((**a**) FeCoNiCrAl powder; (**b**) FeCoNiCrAl coatings).

Figure 5 shows the XRD spectra of HEA powder and coating. The results showed that the sprayed powder was mechanically mixed in equal proportions in the prepared state, and the spectrum could find corresponding elemental diffraction peaks. As shown in Figure 5b, the XRD pattern of the coating prepared at 9 kW is mainly composed of the BCC phase, with a small amount of FCC phase. As the power increases, the intensity of the FCC phase for diffraction peak appearing significantly increases at approximately 45°, and attains its maximum value at 18 kW.

After being heated by a plasma arc with different powers and cooled to form the coating, the phase structure of the HEA coatings transforms into a typical BCC phase structure. There are also significant differences in the phase structure under different spraying powers, and coatings prepared at higher powers generate a small amount of FCC phase. According to the analysis of the phase diagram of high entropy alloys in relevant articles [32], it can be found that the temperature of droplets is also higher after the melting of HEA powder at higher power. During the cooling process, there are multiple phase transition zones, and the rate of temperature drop during the cooling process is higher, making it easier to generate other phases.

Cheng [22] also confirmed this process in their study. The FCC phase is generated by FeCoNiCrAl powder at high temperatures. When the coating is rapidly cooled, some FCC phases fail to transform into BCC phases in a timely manner and remain in the coating. Xu et al. [33] verified Cheng’s conclusion.

Table 2 is the mixing enthalpies for the atomic between elements in FeCoNiCrAl coatings [34]. During plasma spraying, the synergistic diffusion between the main atoms is suppressed due to the high mixing entropy, thereby inhibiting the formation of intermetallic compounds. Then, the coatings form a solid solution in the molten state to be retained after solidification. The phase structure of the coating is a FCC+BCC biphasic solid solution structure, without the formation of complex intermetallic compounds. As shown in Table 2, aluminum has the lowest entropy and the highest chemical activity when mixed with other elements. Therefore, during the spraying process, the mixing of oxygen elements causes aluminum to combine with oxygen to form an oxide form that exists in the coating.

### 3.3. Dynamic Potential Polarization Results

**Figure 6:**
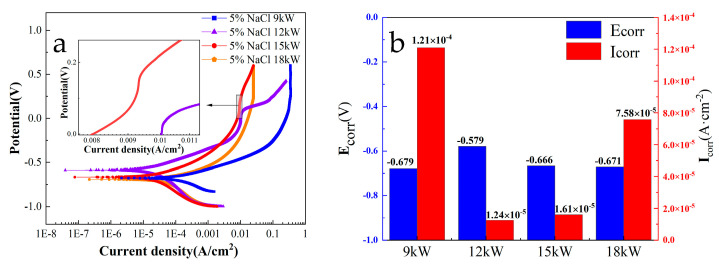
Dynamic potential polarization of FeCoNiCrAl coatings ((**a**) polarization curve; (**b**) result of Tafel fitting).

Figure 6 shows the polarization results of FeCoNiCrAl HEA coatings with different spraying powers at 5 wt.% NaCl solution. The results show that the corrosion potential of each coating was different, and all were between −590~−700 mV. Among them, the coating with a spraying power of 12 kW had the highest corrosion potential of −594 mV. The corrosion current density of each coating varies greatly, and the coating with 9 kW spraying power is higher than the other coatings. The SEM results indicate that the coating with 9 kW spraying power has more defects such as pores and cracks, which accelerates the rate of Cl—penetration and causes the coating corrosion.

During the anodic polarization process of the coating with 12 kW spraying power, the corrosion current density increases with the positive shift in the potential. When the potential reaches −250 mV, with the positive shift in the potential, the anodic current density no longer increases, and passivation occurs. At this time, the components in the coating undergo a passivation reaction under the action of the potential, generating a dense passivation film that hinders the penetration of corrosive ions [35]. When the potential shifts to 30 mV, the corrosion current density of the coating begins to increase again, which indicates that the coating’s passivation layer has been broken down and lost its protective effect. The partial magnification of the polarization curve shows that the coating with 15 kW spraying power also exhibits a passivation phenomenon, but compared with the coating with 12 kW spraying power, the range of passivation zone is smaller and the passivation potential is higher.

Figure 6b shows the fitting results of dynamic potential polarization with different spraying powers at 5 wt.% NaCl solution. The corrosion parameters were obtained by calculating the polarization curve of the dynamic potential using the Tafel fitting method. Usually, an elevated corrosion potential (Ecorr) signifies superior corrosion resistance for the coating, whereas a diminished corrosion current density (Icorr) correlates with a decreased corrosion rate [36]. The results showed that the coating with 12 kW spraying power shows the highest corrosion potential Ecorr and the lowest corrosion current density Icorr.

According to the fitting results of the dynamic potential polarization, the accumulation of oxygen and aluminum during the spraying process substantially affects the coating’s resistance, which not only affects the porosity and corrosion resistance but also has a certain effect on the passivation of the coating. Compared with the four coatings prepared with different power, the coating with 12 kW spraying power shows the best corrosion resistance. Moreover, the passivation effect of the coating prepared at 12 kW is more significant compared to other coatings. This is related to the more uniform distribution of elements on the surface and the lower porosity of the coating.

**Figure 7:**
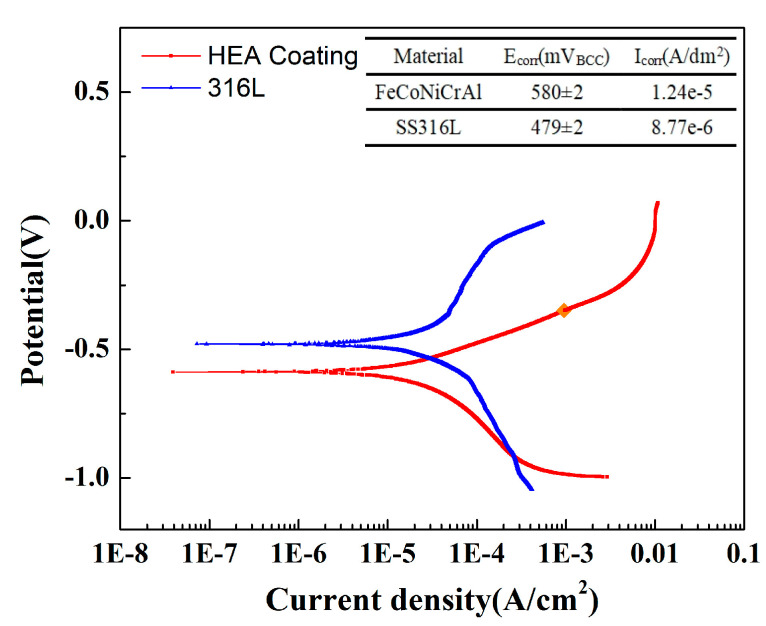
Comparison results of polarization measurement between the HEA coating prepared with 12 kW spraying power and SS316L.

However, the corrosion resistance of HEA coating prepared by air plasma spraying is still lower than that of 316L stainless steel. Figure 7 shows the comparison results of polarization measurement between the HEA coating prepared with 12 kW spraying power and SS316L at 5 wt.% NaCl solution. The corrosion potential of SS316L was significantly higher than that of the HEA coating prepared by air plasma spraying (APS), and the corrosion current density was lower than that of the coating, showing better corrosion resistance. This is mainly because the 316L is more compact than the coating prepared by spraying, and the element distribution is more uniform. There is a certain porosity in the plasma sprayed coating, and the agglomeration of aluminum has a certain impact on the corrosion resistance and passivation process of the material.

### 3.4. EIS Result

**Figure 8:**
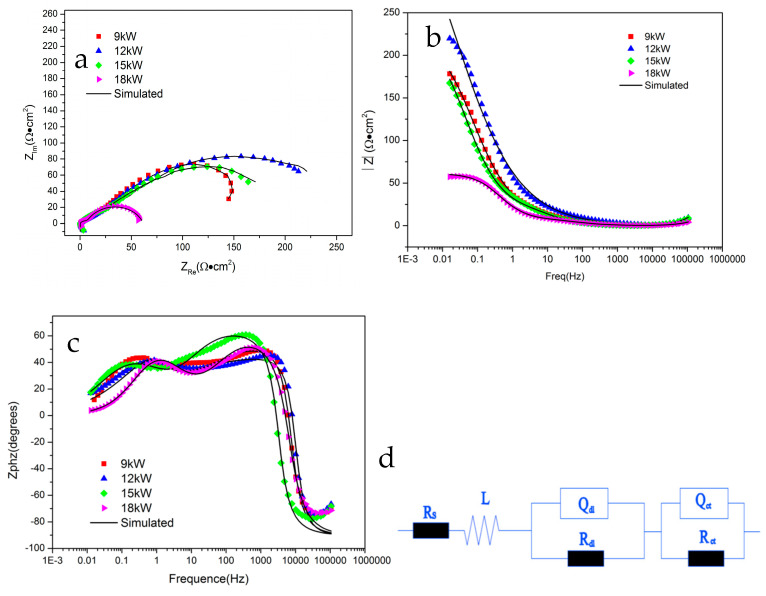
EIS results of FeCoNiCrAl coatings ((**a**) Nyquist plot; (**b**) Bode plots; (**c**) Bode plots; (**d**) equivalent circuit).

Figure 8 shows the Electrochemical Impedance Spectrum of FeCoNiCrAl HEA coating in 5% NaCl solution. Figure 8a–c show the Nyquist plot and Bode plots, and Figure 8d is the corresponding fitting equivalent circuit, and the fitting results obtained from the corresponding circuit components are shown in Table 3.

The Bode plots of all coatings exhibit three time constants. After many times of measurements, the results show that all coatings have negative phase angles in the high-frequency region. This is caused by the influence of the dispersion effect, where the impedance behavior of the solid–liquid interface double layer is not completely consistent with that of the equivalent capacitance, resulting in negative phase angles. The dispersion effect can cause the phase angle to deviate from the normal range and even result in a negative value [37]. Moreover, the stability of the electrode system can also lead to negative high-frequency phase angles, such as unstable passivation or passivation processes on the electrode surface, which can cause excessive potential fluctuations and affect the stability of the entire electrode system, resulting in negative phase angle at the high-frequency region.

At fitting equivalent circuit components, Rs represents the solution resistance, and L represents the inductive element that appears in the high-frequency region. The other two time constants are Qdl, Rdl, and Qct, Rct. Rdl represents the ion diffusion resistance inside the coating. In this equivalent circuit, the diffusion resistance is mainly caused by the resistance of corrosive ions adsorbed by pores and other defects in the coating, as well as the resistance of ion diffusion caused by the passivation film formed with the passivation on the coating surface. Qdl represents the constant phase angle element (CPE) of the corrosive ion diffusion process. CPEs are used in the analysis of impedance spectra, to account for the deviations caused by surface roughness [38,39]. Rct and Qct represent the anodic dissolution resistance of the coating and the phase angle element (CPE) of the anodic process, respectively.

Some research suggests that the occurrence of localized corrosion in AlCoCrFeNi alloy was attributed to the presence of Cr-depleted regions, selective dissolved elements cause localized corrosion and passivation film defects during the corrosion process [40,41]. Moreover, the combination of aluminum and oxygen in the coating causes local element segregation, which also accelerates the corrosion and passivation process. Compared with EIS results, the effect of spraying parameters on coating performance is more obvious, the comparison between Rdl and Rct is 12 kW > 15 kW > 9 kW > 18 kW, which is consistent with the variation law of coating porosity. It shows that the Rdl and Rct of the coating with 12 kW spraying power are the highest among the four coatings, indicating that the coating prepared at 12 kW provides better protection to the substrate, and shows more effect of hindering the corrosion diffusion process, which consistent with the results observed by scanning electron microscopy.

### 3.5. Mott–Schottky Results

To study the passivation process of FeCoNiCrAl HEA coating, Mott–Schottky measurement was conducted. According to the different types of charge carriers inside the semiconductor of the passivation film, the properties of the passivation film are divided into two categories: n-type semiconductors with ion holes as charge carriers; and p-type semiconductors with free ions as charge carriers, respectively [38,39,42].

The point defect density N is expressed as the acceptor concentration NA in p-type semiconductors and as the donor concentration ND in n-type semiconductors. According to the study on the relationship between the point defect density N of alloy passivation film and its properties [43,44], a higher point defect density indicates that the higher the concentration of ion pores and free ions in the passivation film, the faster the adsorption of corrosive ions in the solution, leading to alloy pitting corrosion.

**Figure 9:**
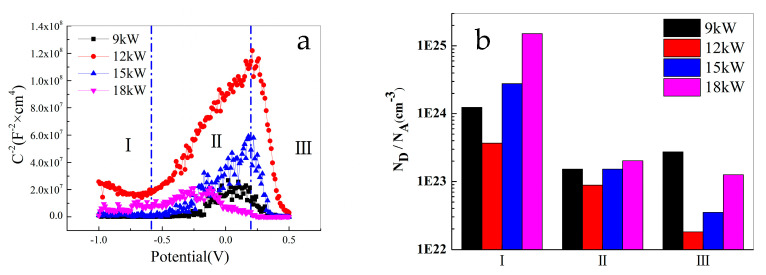
Mott–Schottky results of FeCoNiCrAl coatings ((**a**) M-S curve; (**b**) result of fitting).

Figure 9a shows the performance of FeCoNiCrAl HEA coatings with different spraying powers at 5% NaCl solution, and Figure 9b is the fitting results of the point defect density of the coatings. The M-S results in 5% NaCl solution show three different regions with a positive shift in potential, namely, the semiconductor properties of the passivation film of FeCoNiCrAl HEA coating undergo a transformation in the positive shift in potential. This is because there are many types of elements in the coating and the proportion of elements is the same. During the passivation process, some elements are oxidized to produce oxides with different semiconductor properties, such as Fe_2_O_3_ exhibiting n-type semiconductor properties [45] and Cr_2_O_3_ exhibiting p-type semiconductor properties [46]. At the same time, the priority of oxidation of each element is different, resulting in different contents of various oxides in different periods. Therefore, the semiconductor properties of the coating’s passivation layer vary across distinct regions.

In the range of −1 V~−0.7 V in Zone I, the slope of the M-S curve of the coating with 12 kW spraying power is negative, and those of the other three coatings are close to parallel to the X-axis, the data fluctuations are not significant. It shows that the passivation film of the coating with 12 kW spraying power exhibits p-type semiconductor characteristics. However, compared to the other two zones, the slope of the curve in Zone I is relatively small. This zone corresponds to the cathode region of the polarization curve, which is mainly the passive film property on the surface of the prepared FeCoNiCrAl HEA coating. However, the point defect density of this passivation film is relatively high, and its protective effect on the coating is low. Combined with EDS results, it indicates that the structure of the surface passivation film at this zone should be an oxide film of various elements in the as-prepared coating, showing p-type semiconductor oxides dominating and holes as multi-carrier structures.

In the range of −0.7 V~0.2 V in Zone II, the M-S curve exhibits a positive gradient, and the passivation film of the four coatings exhibits n-type semiconductor characteristics. At this zone, the semiconductor properties of the passivation film of the coating undergo the first transformation, and the slope of the curve sharply increases. This indicates that some unoxidized elements and low-priced oxides in the coating gradually oxidize with a positive change in the potential, including Fe, Cr, Ni, and other elements in the HEA coatings, which exhibit a passivation effect with the positive shift in the potential. At the potential reaches −0.7 V, the main substances in the passivation film at the surface of the films undergo a transformation, thus altering the semiconductor properties of the coating.

The transform potential of the coating with 9 kW spraying power is about 0.1 V; 18 kW is near −0.12 V. The transition potential of the coating with 12 kW, and 15 kW spraying power is close to 0.2 V, which is related to the temperature of the plasma beam and the quality of the coating. At higher power, the coating surface is easier to oxidize, so this transformation is not obvious, and the transform potential is significantly reduced.

In the range of 0.2 V~0.5 V in Zone III, the M-S curve exhibits a negative gradient and the passivation film of the four coatings exhibits p-type semiconductor characteristics. This indicates that when the potential reaches 0.2 V, certain elements and low-priced oxides in the coating reach the conditions for oxidation, generating high-priced oxides. Meanwhile, the passivation film is close to breakdown, and some of the oxides have dissolved, which changes the content of various oxides in the coating. The slope of the curve in Zone III is the largest, indicating that the point defect density of the passivation film in the coating is the smallest, and the performance of the passivation film is the best, which hinders the corrosion of the coating. When the potential exceeds 0.5 V, the M-S curve slope of FeCoNiCrAl HEA coatings with different spraying powers approaches 0, indicating that the passivation film has been broken down and the protective effect on the coating has been lost.

The oxygen combines with the elements of the coating during plasma spraying, which causes the different states of the semiconductor properties. The M-S curves reverse with the potential for the coatings, with the potential increase, the emergence of an additional inversion layer is attributed to the elevated levels of vacancies within the valence band. When the potential is relatively high, the passivation film is damaged by the high potential at the surface of the coating [47,48]. All of those cause p-n type changes in the coating.

Figure 9b shows the fitting results of point defect density. It indicates that the point defect density of the coating with 12 kW spraying power is the lowest, and the protective effect of the passivation film is more obvious than the other coatings, which is related to the more uniform distribution of elements. Comparing the passivation zone of the polarization measurement, the curves of the result have similar shapes. The passivation segment of the polarization measurement appears between −250 mV to 200 mV, which shows an S-shape and is quite similar to those of the M-S measurement.

### 3.6. XPS Results

**Figure 10:**
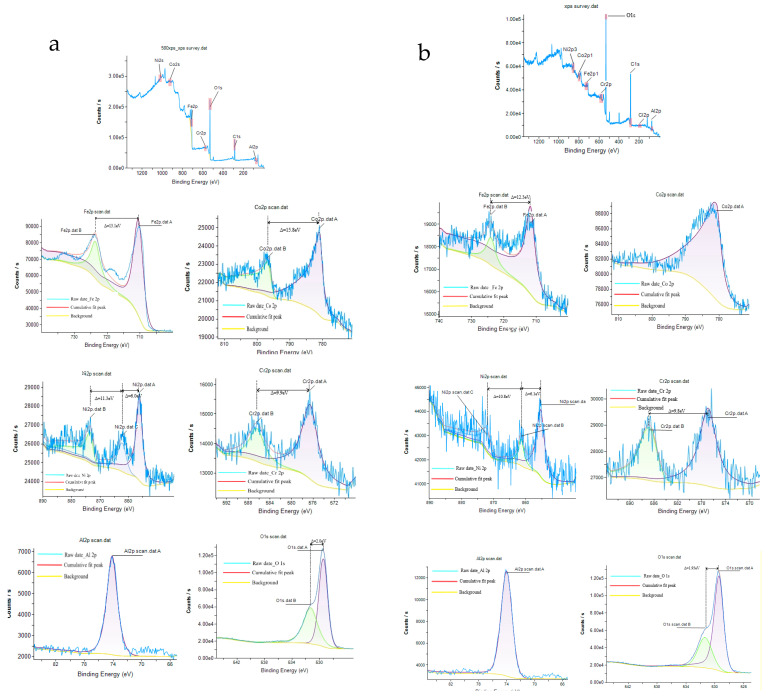
XPS spectra of FeCoNiCrAl HEAs coating ((**a**) as-prepared coating; (**b**) after corrosion).

Figure 10 is the X-ray photoelectron spectroscopy (XPS) spectra of FeCoNiCrAl HEA coating with 12 kW spraying power before and after corrosion ((a) represents as-prepared coating, and (b) represents the coating after corrosion). The results show that most elements in the as-prepared coating exist in the form of oxides, and only a small amount of unoxidized elements are present, which is due to the high temperature during plasma spraying. It encourages each element’s interaction with oxygen and makes the coating contain a certain amount of oxides [37]. However, there are significant differences in the oxidation products generated by the interaction between different elements and oxygen during air plasma spraying, as well as their impact on the corrosion process.

At the as-prepared coating, Iron exists in the form of Fe^3+^ (710.7 eV, 725.2 eV). Most of the Fe elements in the coating have been oxidized to Fe^3+^ during air plasma spraying, and only a small amount of low-priced Iron (718.5 eV, 733.5 eV) exists in the coatings. After corrosion, all of the Irons in the coating are transformed into a high valence state.

As shown in Figure 10, Cobalt appears as Co^2+^ (780.8 eV) in the as-prepared coating, accompanied by a satellite peak (798.6 eV, 803.2 eV). After corrosion, the intensity of satellite peaks in the coating becomes less prominent. The performance of Chromium, Nickel, and aluminum is relatively similar. The main form of Nickel in the as-prepared coating is Ni^2+^ (854.2 eV, 862.3 eV, 873.8 eV), and Chromium is Cr^6+^ (576.5 eV, 586.4 eV). Both of them have a low-priced satellite peak, corresponding to their respective elemental substance. After corrosion, all of them in the coating transform into a high valence state.

Aluminum appears as Al^3+^ (74.2 eV) at the Al 2p3/2 spectrum in the as-prepared coating, and all existed in the form of Al_2_O_3_ without the low-priced satellite peak. The valence state of aluminum indicates that the Al element did not react during the whole corrosion process. After corrosion, the valence state of aluminum remained unchanged, while the intensity of the energy peak significantly decreased. This suggests that aluminum did not undergo passivation during the corrosion process, indicating a phenomenon of preferential corrosion.

The M-S results demonstrated that the passivation films of the coating display P-type semiconductors in the initial stages of corrosion, which correspond to zone I. XPS measurements reveal that the passivation layer is predominantly composed of P-type semiconductors such as Ni^2+^ and Cr^6+^ at this stage [48]. Moreover, Cobalt’s electronic structure, with its fully occupied d orbitals, results in a diminished role in passivation film formation [3]. Conversely, aluminum’s tendency to cluster with oxygen leads to the formation of a robust Al_2_O_3_, hindering the development of a passivation film, the finding supported by SEM results.

As the potential rises, Fe^0^ and low-priced Fe in the coating undergo oxidation to form Fe_2_O_3_, which exhibits characteristics of an N-type semiconductor. Concurrently, Ni^2+^ dissolves with the increase in the potential, which shows a P-type semiconductor, and converts into Ni(OH)_2_. Consequently, the coating’s semiconductor properties shift to N-type, aligning with the findings from zone II of the M-S analysis.

In zone III, the polarization potential exceeds that of other zones. With elevated polarization potential, there is an increased formation of oxygen vacancies and cation interstitials, along with an enhanced rate of their annihilation. This accelerates the passivation film dissolution, contributing significantly to the pitting corrosion observed in the coating. Moreover, the Fe_2_O_3_ is less stable than Cr_2_O_3_ in the film with the increase in potentials, the change in composition, and the potential for passivation film, so it represents the properties of p-type semiconductors.

During the corrosion process, aluminum oxide is difficult to passivate and promotes the formation of local Cr-depleted and Al-rich regions in FeCoNiCrAl HEA coating, which are the reasons for localized corrosion. Compared with other elements, the coating containing about 20 mol% of aluminum is more likely to cause local pitting corrosion in the coating [49].

**Figure 11:**
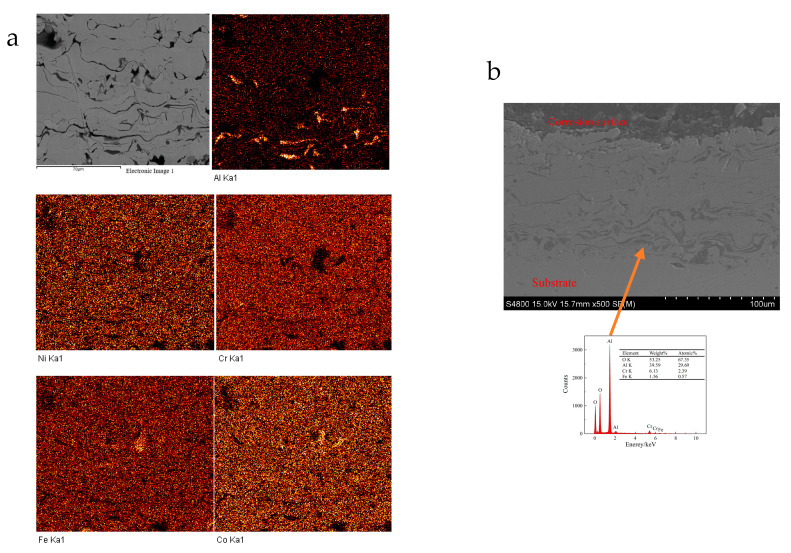
Element distribution mappings of the coating at cross-sectional scanning after corrosion ((**a**) element distribution mappings; (**b**) sectional image and partial EDS results).

The mappings of element distribution for the coating at cross-sectional scanning and partial EDS results after corrosion are shown in Figure 11. The section images show that the connection between the coating and the substrate is good without obvious change. Therefore, the corrosion is mainly for the coating, and the aluminum-rich phase in the coating is the key factor causing the corrosion of the coating. The element distribution mapping result indicates that the aluminum concentration near the outer surface of the coating was significantly lower than that at the inner surface. The distribution of aluminum in the coating increased with the depth of the coating, and the distribution of other elements was relatively uniform. Compared with the line-scanning results of the as-prepared coating, aluminum oxide in the coating is more prone to corrosion during the corrosion process, aluminum and aluminum oxide near the outer surface of the coating are more likely to corrode into the solution, causing localized corrosion, and ultimately leading to uneven distribution of aluminum concentration. The concentration distribution of elements in the solution after corrosion is shown in Table 4. It also indicates that the concentration of aluminum ions in the solution is significantly higher than that of others, while the concentration of Chromium ions is slightly lower, which suggests that the phenomenon of preferential corrosion occurs in the Cr-depleted and Al-rich regions. This is also the reason that the corrosion resistance of FeCoNiCrAl HEA coating prepared by air plasma sprayed is lower than that of SS316L.

## 4. Conclusions

FeCoNiCrAl HEA coatings were prepared by air plasma spraying, and the corrosion resistance and passivation effect of the coatings prepared at different powers were studied.

(1) The coating prepared with the optimal power for spraying parameters has the best quality. The research results indicate that the coating prepared with 12 kW spraying power has the advantages of low porosity and uniform element distribution, which leads to its good corrosion resistance. The coating prepared with 12 kW spraying power is mainly composed of the BCC phase, with a small amount of FCC phase;
(2) The molten droplets formed during the preparation process of HEA coatings easily combined with oxygen, among which aluminum and oxygen are more easily combined and exist in the form of aluminum oxide in the coating, while other elements have weaker binding with oxygen, ultimately forming Cr-depleted and Al-rich regions;
(3) The passivation state of FeCoNiCrAl HEA coatings changes with potential and the M-S curve shows an S-shaped transition, indicating that various elements in the coating undergo the p-n performance transformation processes of the passivation film during potential changes. Among them, the passivation effect of the coating prepared with 12 kW spraying power is more obvious, and the density of point defects in the passivation film is the lowest;
(4) The XPS results indicate that there are high valence states and a small amount of low valence states for Fe/Co/Ni/Cr in the as-prepared coatings. After corrosion, they all transform into stable high valence states, which is consistent with the MS results. Aluminum forms a relatively stable compound with oxygen in the as-prepared coatings, which is not easily passivated. Moreover, Cr-depleted and Al-rich regions in the coating preferentially corrode, which is the cause of localized corrosion during the corrosion process.

## Figures and Tables

**Table 1:** Process parameters of plasma spraying.

Power (KW)	Current (A)	Voltage (V)	Spraying Distance (mm)	Ar Flow Rate (L·min^−1^)	Powder Feeding (g·min^−1^)
9	300	30	100	100	30
12	400	30	100	100	30
15	500	30	100	100	30
18	600	30	100	100	30

**Table 2:** Mixing enthalpies for atomic between elements in FeCoNiCrAl coatings (KJ/mol).

Element	Fe	Co	Ni	Cr	Al
Fe	-	−1	−2	−1	−11
Co	−1	-	0	−4	−19
Ni	−2	0	-	−7	−22
Cr	−1	−4	−7	-	−10
Al	−11	−19	−22	−10	-

**Table 3:** Fitting result of EIS spectrum.

	9 kW	12 kW	15 kW	18 kW
Rs	0.155	0.187	0.165	0.129
L	9.53 × 10^−6^	1.52 × 10^−5^	1.46 × 10^−5^	6.63 × 10^−6^
CPE	0.0091	0.0126	0.0136	0.0093
F	0.68	0.63	0.69	0.75
Rdl	102	308.3	216	67.13
CPE	0.0033	0.0061	0.0027	0.01076
F	0.63	0.45	0.77	0.81
Rct	110.3	233.5	117.21	54.68

**Table 4:** Element dissolution in corrosion solution by using inductively coupled plasma optical emission spectroscopy.

Measurement	Fe	Co	Ni	Cr	Al
Result(ppm)	118	88	98	55	229

## Data Availability

The original contributions presented in this study are included in the article/Supplementary Materials. Further inquiries can be directed to the corresponding authors.

## Supplementary Materials

The following supporting information can be downloaded at: https://www.mdpi.com/article/10.3390/ma18071396/s1, (1) The power images; (2): Table: Porosity results of the coatings of FeCoNiCrAl HEAs.
