# Supplementary material for: Study on the Process Parameters and Corrosion Resistance of FeCoNiCrAl High Entropy Alloy Coating Prepared by Atmospheric Plasma Spraying
Source: Materials (Basel). 2025 Mar 21;18(7):1396. doi: 10.3390/ma18071396 (PMC11989591; doi:10.3390/ma18071396)
Supplement: Supplementary file 1 [file materials-18-01396-s001.zip › materials-3499643-supplementary.pdf]

Supplementary files

(1) The power images

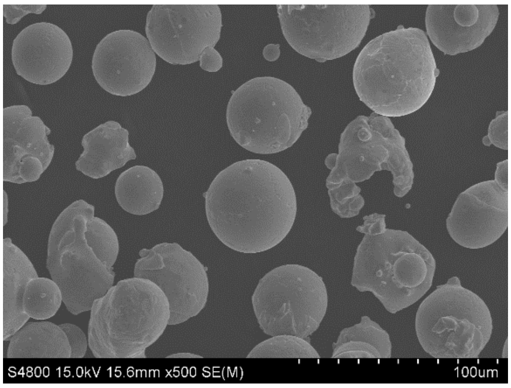

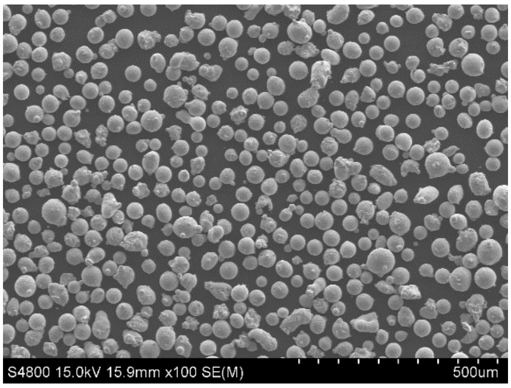

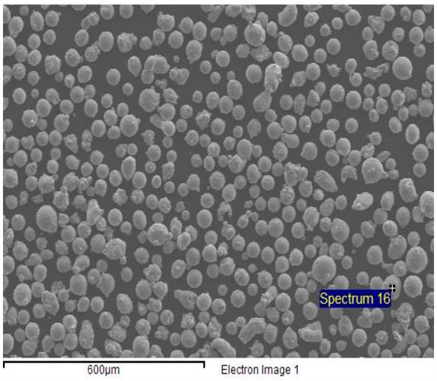

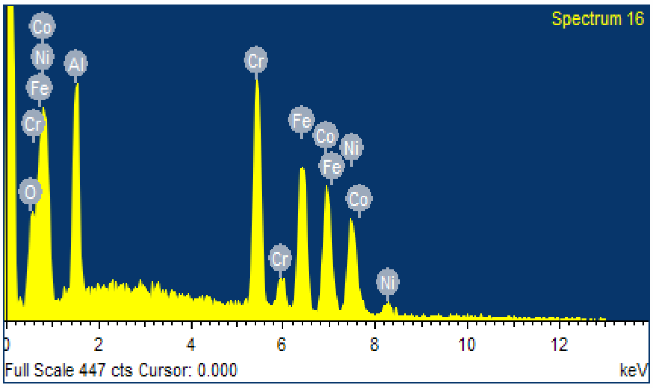

Element	Weight%	Atomic%
O K	2.01	6.22
Al K	8.11	14.85
Cr K	21.59	20.53
Fe K	22.03	19.51
Co K	23.79	19.96
Ni K	22.48	18.93
Totals	100.00	

(2) Table: Porosity results of the coatings of FeCoNiCrAl HEAs

Power	9kW	12kW	15kW	18kW
Porosity	3.4	2.6	3.1	4.3
